# Do government environmental audits reduce air pollution? Evidence obtained from Lanzhou

**DOI:** 10.3389/fpubh.2024.1470112

**Published:** 2024-11-06

**Authors:** Tianyou Chen, Ji Li

**Affiliations:** ^1^China Center for Special Economic Zone Research, Shenzhen University, Shenzhen, China; ^2^Research and Development Office, Shenzhen Polytechnic University, Shenzhen, China

**Keywords:** government environmental audit, air quality, regression discontinuity design, environmental protection policies, air pollution

## Abstract

**Introduction:**

This study investigates the effectiveness of government environmental audits in mitigating air pollution. Specifically, it focuses on a pilot project conducted in Lanzhou City by the former Ministry of Environmental Protection. The research employs a regression discontinuity design to empirically assess the impact of these audits on air quality.

**Methods:**

The study utilizes a regression discontinuity design to evaluate the impact of the government environmental audits. The primary outcome variable is the Air Quality Index (AQI). The analysis includes extensive robustness checks, such as sensitivity testing for optimal bandwidths of key pollutants, adjusting for precipitation levels, and conducting various robustness tests to ensure the reliability of the results.

**Results:**

The findings reveal a significant decline in the AQI following the implementation of the environmental audits. The AQI decreased from 31 to 25 during and after the audit period. Additionally, the study observed a notable increase in precipitation levels, suggesting potential environmental improvements. Robustness tests further confirm the reliability of the regression results.

**Discussion:**

The research outcomes demonstrate the effectiveness of government environmental audits in reducing air pollution. By supervising local governments in the implementation of environmental protection policies, these audits contribute to improved air quality. The findings highlight the importance of targeted interventions and policy enforcement in addressing environmental challenges.

## 1 Introduction

China has achieved unprecedented economic development since it instituted reforms and opened up. It has realized a historical transformation from a poor and backward agricultural country to the world’s leading industrial manufacturing nation, attaining success that attracts worldwide attention. However, the long-term impact of the extensive economic development mode has resulted in serious air pollution emanating from the discharge of numerous harmful gasses and particles such as industrial exhaust gas, automobile exhaust gas, and industrial dust into the atmosphere without due treatment. In 2011, China became the country emitting the highest amounts of greenhouse gasses in the world. China’s industrial exhaust gas emissions expanded to 67450.93 billion standard cubic meters, 1.84 times higher than the levels noted in 2004. Serious air pollution can cause frequent occurrences of adverse weather conditions such as haze and acid rain; it can also trigger human respiratory diseases such as asthma (1). Furthermore, air pollution severely damages the health of citizens (2) and exerts a negative impact on economic growth (3). The issue of air pollution has become a focal concern for the public over the past few years. Chinese governments at all levels have also realized the gravity of air pollution and are actively attempting to tackle this problem.

As early as 2013, the State Council issued the Action Plan for Prevention and Control of Air Pollution. This policy clearly showed that by 2017, the concentration of inhalable particulate matter would be reduced by more than 10% of the levels registered in 2012 in cities at the prefecture level and above, thus improving the overall air quality of the country and greatly reducing weather conditions triggered by heavy pollution (4). In January 2014, the Ministry of Environmental Protection asked 31 provinces, municipalities, and autonomous regions to sign a letter of responsibility for air pollution prevention and outline control objectives. This document defined the objectives and key tasks of local air quality improvement and assigned further environmental protection responsibilities to local governments. In 2018, the State Council issued and implemented a three-year action plan to win the war of protecting blue skies. This strategy indicated that the total amounts of major air pollutants would be significantly reduced after 3 years of effort. Greenhouse gas emissions would be decreased in a coordinated manner, and the concentration of fine particulate matter (PM_2.5_) would further be significantly lowered to promote continuous improvements in ambient air quality. The bid to control air pollution cannot be separated from effective supervision. The government environmental audit denotes a prominent aspect of national governance. It can unquestionably deliver effective supervision to regulate air pollution.

The National Audit Office separately listed the environmental audit in its Audit Work Development Plan from 2008 to 2012. This plan preliminarily defined the objects and scope of the environmental audit; furthermore, it highlighted the need for efforts to build an environmental audit model conforming to the actual conditions prevailing in China. In 2009, the National Audit Office issued the opinions on strengthening the audit of resources and environment, which put forward specific requirements on the main tasks, work systems, theoretical research, and other aspects of environmental audits. This document became a guideline for the promotion and execution of resource and environment audits by nationwide audit institutions of that time (5). However, the government environmental audit is a brand-new product that differs from the traditional financial audit. No ready-made example exists to direct its operations. There is also no way to apprehend the effects of its bid to control environmental pollution. In 2015, the Ministry of Environmental Protection executed the government environmental audit pilot in Lanzhou City in Gansu Province, seeking to accrue the experience to design a government environmental audit system. The inclusion of environmental auditing in the development plan by the National Audit Office is an important measure to respond to the construction of national ecological civilization and promote green and low-carbon development. Worldwide, environmental auditing has become an important component of government auditing, serving as a crucial tool for evaluating the effectiveness of environmental protection policies, promoting efficient resource utilization, and environmental protection. Through environmental auditing, it is possible to strengthen supervision over the use of environmental protection funds and the implementation of environmental policies, ensuring that environmental protection funds are used exclusively and policies are implemented effectively. Reveal the problems in environmental management, promote the rectification and implementation of audited units, and enhance their environmental governance capabilities. The openness and transparency of environmental audit results can help enhance public awareness of environmental protection and create a good atmosphere for the whole society to participate in environmental protection. With the increasingly severe global environmental problems, environmental auditing, as an important means of environmental supervision, plays an increasingly prominent role in national governance and sustainable development.

Lanzhou, located in northwest China within the Lanzhou Basin, faces significant environmental challenges primarily due to industrial emissions, coal-fired heating systems, and increasing vehicular exhaust. This unique geographical setting, characterized by mountains and a basin topography, traps air pollutants, leading to poor air quality exacerbated by low wind speeds and minimal rainfall—averaging less than 300 mm annually. Recognizing the urgency of its environmental issues, the Lanzhou Municipal Government has intensified its commitment to environmental auditing and governance. In recent years, it has implemented rigorous auditing methodologies to identify and address inefficiencies in environmental management. These initiatives aim to not only resolve immediate pollution challenges but also foster systemic improvements in governance.

In response to the critical air pollution situation, Lanzhou formulated the Air Pollution Prevention and Control Action Plan, significantly enhancing local air quality in recent years. Being the first city to pilot the government environmental audit, Lanzhou actively promotes measures to monitor and control air quality, focusing on the municipal government’s implementation of the action plan. Through these efforts, Lanzhou is shifting toward a proactive approach to environmental management, emphasizing prevention, accountability, and continuous improvement, ultimately paving the way for a cleaner and more sustainable urban environment for its residents.

This study focuses on the investigation background of environmental audits conducted by the Lanzhou Municipal Government in recent years, and collects and analyzes relevant data. Evaluate the actual effectiveness of government environmental audits in improving air quality, in order to provide valuable references for future environmental governance.

Three principal methods are currently applied to test the effects of policy implementation. First, the ordinary least square method merely allows researchers to compare changes in air quality before and after a government environmental audit is implemented. Second, the difference-in-difference technique involves the selection of a target area and the creation of a control group comprising other cities. Variations in the targeted area are observed during the implementation of the government environmental audit and the times when the government environmental audit is not in effect. In addition, disparities are noted between cities in which government environmental audits are or are not conducted. Third, the regression discontinuity method allows researchers to observe whether a sudden change occurs in air quality after the implementation of a government environmental audit.

In terms of disadvantages, the ordinary least square method cannot distinguish the effects of the government environmental audit from the impact of other policies or identify the inherent trends of air quality change in the target area, in this case, Lanzhou. The difference-in-difference technique cannot be applied in the case of distinctive cities such as Lanzhou. Just as Mexico City is a very special city in Mexico (26), Lanzhou is also a very unusual city in China. It is impossible to find a city in China that mirrors or resembles Lanzhou because of its unique location, terrain, industries, and other factors. Thus, a matching control group cannot be established. Further, the difference-in-difference method cannot distinguish the influence of a government environmental audit on air quality in Lanzhou from the effects of other policies. In comparison to the ordinary least square and difference-in-difference methods, the regression discontinuity approach can better resolve the problem of identifying the impact of a government environmental audit. Regression discontinuity offers scholars the advantage of being able to distinguish the effects of running variables from the impact of other, continuously changing variables to accurately estimate the effects of the running variables on changes in air quality.

In response to the specific situation in Lanzhou City, this study innovatively adopted the econometric method of regression discontinuity design. This method effectively controlled the interference of other potential influencing factors by simulating the conditions of random experiments, making the evaluation of the environmental audit effect of the Lanzhou government more accurate and reliable. This study assumes that the implementation of government environmental audits can significantly improve the efficiency of air pollution control in Lanzhou City and promote sustained improvement in air quality. Through empirical analysis, we found that the air quality in Lanzhou City has significantly improved after receiving government environmental audits, and this improvement has not rebounded, which fully verifies the positive role of government environmental audits in promoting air pollution control. This discovery not only proves the effective implementation of the Lanzhou Air Pollution Prevention and Control Action Plan, but also provides valuable policy references for other cities facing similar contradictions between economic development and environmental protection.

## 2 Literature review

### 2.1 Theoretical analysis of environmental audit

Fan and Huang (6) conducted an in-depth analysis of the challenges faced by Japan in flood risk management in response to climate change. Not only does it reveal profound changes in the nature of hazards, exposure levels, and vulnerability, but it also focuses on the new strategies innovatively developed by the Japanese government to address these challenges, especially those measures to strengthen flood risk management through the latest legal framework. Maltby (7) described environmental audits as activities appraising the environmental management compliance of audited entities with the relevant environmental laws and regulations and as the postulation of suggestions for improvement. Todea et al. (27) listed and described several definitions of environmental audits mooted by organizations such as the International Chamber of Commerce, the Council of the European Union, ISO14050, and the Eco-Management and Audit Scheme. They found that these definitions emphasized discrete aspects. Todea et al. (27) opined that the environmental audit denotes a systematic analysis of the environmental impact of enterprises. Xie et al. (8) asserted that environmental audits conducted by auditing institutions serve as a form of economic supervision distinct from administrative and technical oversight. Several scholars have explored not only the concepts underlying environmental audits but also their criteria, implementation pathways, and content. Zhan et al. (9) proposed the establishment of an environmental early warning system. Chen et al. (10) initiated a discussion on the relevant aspects of China’s government environmental auditing standards, particularly in relation to contemporary changes in international and national policies, regulations, auditing standards, and environmental protection accounting principles. They recommend the prompt establishment of government environmental auditing standards. Leng et al. (11) also offered preliminary recommendations for developing a theoretical framework for these standards. Zhang et al. (12, 13) approached the topic from the perspective of big data within the context of government environmental governance systems, proposing a pathway for implementing government environmental accountability audits. Wang (28) outlined relevant tasks related to resources and environmental audits, focusing on four aspects: resource development and utilization, environmental protection, ecological construction, and climate change adaptation. Furthermore, scholars such as Guo (5), Xu and Chen (14), and Wu and Guo (15) have conducted in-depth research on the content of government environmental audits, providing valuable suggestions for the development of environmental auditing practices.

The aforementioned literature may be classified as normative research that primarily discussed the definition of a government environmental audit. Furthermore, previous studies have discussed the system, contents, and methods of government environmental audits and summarized the practical experiences of Chinese and foreign government environmental audits. Such studies have indicated the need for China to implement government environmental audits and postulated methodologies applicable to China given the outcomes of the existing research. The extant literature also comprises research on prevailing problems and proposes solutions for government environmental audits. The research conducted thus far has derived generally similar conclusions.

### 2.2 Empirical analysis of environmental audit

Empirical research conducted by Li et al. (16) found that government environmental audits facilitated improvements in environmental performance. Cai et al. (17) empirically studied the impact of government environmental audits on corporate environmental information disclosures in the context of the “Three Rivers and Three Lakes” environmental audit implemented by the National Audit Office. They found that the government environmental audit executed by the National Audit Office significantly enhanced the level and quality of corporate environmental information disclosure and stimulated enterprises to fulfill their responsibility of disclosing environmental information. Yu et al. (18) took the *Opinions of the Audit Office on Strengthening the Audit of Resources and Environment* issued in 2009 as an event, subsequently designing a quasi-natural experiment and using the difference-in-difference methodology to examine the impact of government environmental audits on corporate environmental performance. They found that government environmental audits markedly and positively influenced corporate environmental performance. The establishment and implementation of these systems help regulate the environmental behavior of enterprises and improve their environmental management level. Government environmental audits may promote enterprises to optimize their industrial structure, eliminate high polluting and high energy consuming outdated production capacity, and shift toward green and low-carbon production methods. At the same time, the review and evaluation of environmental protection technologies by enterprises during the audit process may also incentivize them to innovate and develop more environmentally friendly and efficient production technologies, thereby improving their environmental performance. Furthermore, Yu et al. (19) employed the super-efficient DEA model to conduct an empirical analysis of the relationships between government audits and air pollution control efficiency. Their results revealed that government audits can promote the efficiency of air pollution control.

However, most current literature comprises studies categorized as qualitative research. Scant quantitative investigations have been conducted to date on the relationships between government environmental audits and environmental pollution governance. The existing empirical research results remain insufficient and incomplete and cannot comprehensively reveal the mechanisms and implementation effects of government environmental audits. Thus, empirical research initiatives must be further extended and intensified. Based on the actual needs of environmental protection work, establish a scientific, reasonable, and comprehensive environmental audit index system, including resource utilization efficiency, pollutant emission control, ecological environment protection, and other aspects. Drawing on advanced international environmental audit standards and experience, and combining with China’s national conditions, we will develop environmental audit standards that are in line with international standards, and enhance the internationalization level of China’s environmental audit work. Establish an environmental audit performance evaluation mechanism, regularly evaluate and provide feedback on the results of environmental audit work, promptly identify problems, and take measures to improve. Strengthen the professional training of environmental auditors to enhance their professional competence and comprehensive abilities in environmental science, environmental management, environmental law, and other areas. Actively introduce professional talents in the fields of environmental science, environmental engineering, environmental law, etc., enrich the environmental audit team, and enhance the overall quality of the team. Establish a sound incentive mechanism for environmental auditors, encourage them to actively participate in environmental audit work, and enhance their work enthusiasm and creativity.

## 3 Theoretical analysis and research hypothesis

The present study takes the perspective of public accountability and considers the government audit as a public appraisal task that the audit institutions accept the entrustment of the people and provides the masses with the performance of government public responsibilities. The government environmental audit conducted by the Central Environmental Protection Department can guide the follow-up audit of local governments’ implementation of central environmental protection policies. And it can correct potentially ineffective execution situations. Operationally, audit institutions can inspect the policies and measures adopted by local governments in terms of pollutant emissions, industrial structure, and energy structure adjustment. They can also examine the use, allocation, and flow of environmental protection funds for local governments. Compared to other regulatory measures, the objects of government environmental audits are not limited to local governments and relevant functional departments. Such audits can also directly intervene in enterprises engaged in polluting industries. The government environmental audit is all-embracing for enterprises; they can include the use of environmental protection funds for enterprises and check whether enterprises have installed adequate environmental protection equipment to comply with requirements. In addition, the government environmental audit can access the environmental monitoring data for the area in which the audited enterprises are located to scrutinize the pollutant emissions of enterprises. The production and operation activities of enterprises in polluting industries significantly impact the social and ecological environment. Enterprise operators tend to take the initiative to adopt environmental protection measures to reduce pollution emissions when auditing institutions conduct government environmental audits on such enterprises and they are directly pressured to follow environmental protection policies. Any problems with the audited enterprises are revealed during the inspection. Once such problems are revealed, other enterprises engaged in polluting industries are also deterred by the prospect of an environmental audit and actively follow through to strengthen their environmental protection management. Apart from conducting routine inspections of local governments and enterprises, audit institutions should also closely attend to public opinion, exhibit empathy with complaints, carefully register information reported by the masses, and promptly stop any violations of laws and regulations. The present study has concluded thus far that government environmental audits can help to continuously and steadily improve the air quality of Lanzhou City by undertaking environmental audits of the Lanzhou Municipal Government and Lanzhou-based enterprises.

To thoroughly investigate the impact of government environmental audits on air quality improvement in Lanzhou, we hypothesize that these audits can significantly enhance the region’s air quality. Therefore, this study adopted the sharp regression discontinuity model to test the effects of the government environmental audit on air pollution control in Lanzhou. The specific model was set as follows:

$\begin{array}{l} y_{t}=\alpha_{0}+\alpha_{1}{\mathrm{GovEnvrAudit}}_{t}+\alpha_{2}\;f\left(T\right)\\ \;\;\;\;\;\;\;+\alpha_{3}{\mathrm{GovEnvrAudit}}_{t}\;f\left(T\right)+\lambda X_{t}+ut. \end{array}$

The explained variable y in this model denoted the air quality index (AQI) along with the daily data of individual pollutant indexes including fine particulate matter (PM_2.5_), inhalable particulate matter (PM_10_), carbon monoxide (CO), nitrogen dioxide (NO_2_), ozone (O_3_), and sulfur dioxide (SO_2_). CO was measured using mg/m^3^ as the unit, whereas the other five single pollutants were measured in *μ* G/m^3^; the subscript t indicated the date corresponding to the data. The explanatory variable GovEnvrAudi was a virtual variable, indicating the government environmental audit. The regression coefficient α_1_ signified the improvement effect of the government environmental audit on air quality (20). T symbolized the running variable, denoting the number of days elapsing after the official implementation of the government environmental audit. The Ministry of Environmental Protection issued the notice launching the government environmental audit pilot on February 15, 2015. The present study created an accurate discontinuity by taking February 15, 2015, as the boundary. The value was set to 0 on February 15, 2015, and estimated to be negative before and positive after that date. The f (T) represented the polynomial of T, and the seventh-order term for it was selected for this study according to the local change trend characteristics of each pollutant. X symbolized a group of control variables that affect air quality, including the daily maximum temperature, daily minimum temperature, wind force, holidays, and the occurrence or absence of precipitation.

## 4 Data and statistical analysis

### 4.1 Data

The AQI from January 1, 2014 to June 30, 2016 was established as the explanatory variable in this study along with the daily average concentration data of six pollutants including CO, NO_2_, PM_10_, PM_2.5_, O_3_, and SO_2_ which were used to monitor the air quality over the same period. The AQI represented the main explanatory variable, and the data pertaining to individual pollutants were also considered. The single conceptual indicator AQI integrates the concentrations of six conventional monitored pollutants. The AQI marks ambient air quality, ranging in value from 0 to 500; the higher the AQI value is, the more serious the pollution is and the greater the harm to the human body is. The data on air quality were collected from *Tianqihoubao*.^1^ Furthermore, the data obtained from this website were extremely consistent with the official data. Therefore, the data sample evinced superior confidence levels.

Natural factors could impact urban air quality (21). Therefore, the present study collected as many control variables as possible for weather conditions, including the daily maximum temperature (h_temp, degree Celsius), daily minimum temperature (l_temp, degree Celsius), wind level (wind), and the occurrence or absence of precipitation (rain). Among these variables, the rain was set as a dummy element in this model, and the value rule of the rain was established as 1 when precipitation occurred and 0 when there was no precipitation. The data of these meteorological variables were also obtained from the website *Tianqihoubao*.

Air quality could also be related to holidays; hence, this study also controlled the variables of holidays. Production activities are undertaken more on working days, causing increased pollution emissions; similarly, production activities decrease during holidays, resulting in reduced pollution emissions. The holiday variables set for this study included legal leaves and weekends, which were categorized according to the holiday arrangement notice issued by the general office of the State Council. A holiday variable labeled “holiday” was thus established for this study. The value 1 was assigned for weekends or legal holidays and 0 for weekdays.

### 4.2 Statistical analysis

The statistical characteristics of the main variables are displayed in Table 1.

**Table 1:** Descriptive statistics.

Variable	Description	Obs	Mean	Std. Dev.	Min	Max
AQI	Air quality index	908	89.684	36.637	29	382
PM_2.5_	Fine particulate matter	908	51.552	25.886	12	292
PM_10_	Inhalable particulate matter	908	117.263	67	22	977
So_2_	Sulfur dioxide	908	21.187	12.667	3	75
No_2_	Nitrogen dioxide	908	44.394	16.41	11	114
Co	Carbon monoxide	908	1.34	0.649	0.46	4.25
O_3_	Ozone	908	47.209	18.975	7	140
h_temp	Daily maximum temperature	908	17.664	10.1	−7	38
l_temp	Daily minimum temperature	908	5.567	9.109	−17	23
Wind	Wind force	908	3.064	0.245	3	4
Rain	Precipitation	908	0.219	0.414	0	1
Holiday	Statutory holidays including weekends	908	0.319	0.446	0	1

The above statistics are based on daily data. Precipitation and statutory holidays are dummy variables. Wind force values range between 3 and 4 according to the wind strength reported on that day.

## 5 Empirical results and analysis

### 5.1 Overall improvement in air quality index

According to the regression discontinuity results in Table 2, the implementation of government environmental audits has had a significant negative impact on the AQI of Lanzhou City, which directly reflects the effectiveness of audit actions in improving overall air quality. After controlling for the variables such as weather conditions and holiday effects, this result is particularly significant, indicating that government environmental audits are an effective means of improving air quality.

**Table 2:** Results of regression discontinuity.

	(1)	(2)	(3)	(4)	(5)	(6)	(7)
Variable	AQI	PM_25_	PM_10_	SO_2_	NO_2_	CO	O_3_
	−59.64**	−37.25***	−90.73**	−18.60***	−27.31***	−0.730**	25.54***
	(29.0452)	(13.2026)	(43.2939)	(6.4465)	(6.8763)	(0.2912)	(4.0542)
Controlling variables	Yes	Yes	Yes	Yes	Yes	Yes	Yes
*N*	908	908	908	908	908	908	908

The polynomial of all the controlling variables selects the 7th-degree term; Standard errors in parentheses; ***, **, or * indicates significance at 1, 5, and 10% levels, respectively.

### 5.2 The concentration of major pollutants has significantly decreased

Further analysis indicates that government environmental audits have a substantial inhibitory effect on key air pollutants (Table 2). Specifically, the concentrations of PM_2.5_ and PM_10_, which represent fine particulate matter and inhalable particulate matter respectively, have demonstrated a notable decline following the implementation of the audit. This discovery validates the effectiveness of audit measures in reducing particulate matter emissions, improving atmospheric visibility, and reducing health risks. As typical atmospheric pollutants, the concentrations of sulfur dioxide (SO_2_) and nitrogen dioxide (NO_2_) have also significantly decreased after auditing. This indicates that government environmental audits not only focus on controlling particulate matter, but also effectively promote the reduction of harmful gasses in industrial emissions sources and other pollution sources. The decrease in carbon monoxide (CO) concentration also indicates the effectiveness of audit measures in regulating traffic emissions and incomplete combustion processes. This helps to reduce health problems caused by CO exposure.

### 5.3 Special analysis of O_3_ concentration changes

Although government environmental audits have generally promoted the improvement of air quality, the regression results regarding ozone (O_3_) concentration present a complex phenomenon. The concentration of O_3_ did not decrease as expected, but instead showed a positive correlation with government environmental audits. This phenomenon can be explained from the following aspects. O_3_ is a secondary pollutant. Its generation is complex influenced by various precursor pollutants (such as nitrogen oxides, volatile organic compounds) and meteorological conditions (such as temperature, light). Therefore, even if the emissions of precursor pollutants are controlled, the concentration of O_3_ may fluctuate due to changes in meteorological conditions.

Indirect effects of auditing: Government environmental audits may primarily focus on reducing the emissions of precursor pollutants. And these emission reduction measures may promote the conversion of remaining precursor pollutants into more O_3_ under specific conditions, such as high temperature and strong light exposure. In addition, meteorological conditions during the audit implementation period may also be a key factor affecting changes in O_3_ concentration.

The complexity of meteorological factors: Wind speed, humidity, and other meteorological factors also play an important role in the diffusion and transportation of O_3_. If the meteorological conditions during the audit period are favorable for the generation of O_3_ but unfavorable for its diffusion, it may lead to an increase in the observed O_3_ concentration.

### 5.4 Bandwidth adjustment

In Regression Discontinuous Design (RDD), the selection of bandwidth is a crucial step as it directly affects the accuracy and robustness of the estimation results. The bandwidth determines the range of observations included in the regression analysis near the cutoff point, and a bandwidth that is too wide or too narrow can lead to estimation bias. Select the bandwidth that minimizes the prediction error through methods such as cross validation. However, in RDD, this method may not be fully applicable as we focus on the processing effects at cutoff point. Lee and Lemieux (22) stated that the bandwidth could affect the robustness of the results of RDD. Hence, a robustness test was mandated for the bandwidth. The default bandwidth calculation was “mserd” and it was selected for the present study’s regression discontinuity model (23). The robustness test was conducted with the optimal bandwidths of the AQI and six pollutants as obtained through the mserd calculation. The seventh-order polynomial was still selected and the optimal bandwidths were, respectively, reduced and increased by 20 and 40 for each explained variable. The regression discontinuity results presented in Table 3 were obtained after the bandwidth adjustment. These findings revealed that the AQI and the concentrations of PM_10_, PM_2.5_, SO_2_, NO_2_, and CO continued to display downward trends when different bandwidths were selected, while the O_3_ concentration trend remained upward (Table 3). In congruence with the previously derived conclusions, these results indicated an amelioration in overall air quality and further supported the outcomes of the benchmark regression.

**Table 3:** Results of bandwidth adjustment.

Variables	AQI	PM_2.5_	PM_10_	SO_2_	NO_2_	CO	O_3_
Optimal bandwidth	254.874	219.762	244.033	216.475	197.514	216.022	199.422
Bandwidth −20	−62.65**	−37.02***	−90.03*	−21.36***	−30.00***	−0.864***	29.04***
	(31.2352)	(13.9579)	(46.7024)	(6.8427)	(7.3732)	(0.3071)	(4.2692)
	234.874	199.762	224.033	196.475	177.514	196.022	179.422
Bandwidth −40	−62.57*	−33.07**	−85.83*	−23.60***	−25.90***	−1.038***	23.58***
	(33.6768)	(14.6379)	(50.5254)	(7.3329)	(7.9210)	(0.3293)	(4.4384)
	214.874	179.762	204.033	176.475	157.514	176.022	159.422
Bandwidth +20	−55.40**	−35.08***	−87.76**	−15.09**	−25.09***	−0.640**	24.18***
	(27.1427)	(12.5516)	(40.4291)	(6.1099)	(6.5421)	(0.2792)	(3.9254)
	274.874	239.762	264.033	236.475	217.514	236.022	219.422
Bandwidth +40	−48.58*	−34.31***	−80.76**	−12.93**	−23.59***	−0.642**	23.79***
	(25.2648)	(11.9653)	(37.6266)	(5.8244)	(6.2472)	(0.2683)	(3.8603)
	294.874	259.762	284.033	256.475	237.514	256.022	239.422
Controlling variables	Yes	Yes	Yes	Yes	Yes	Yes	Yes
*N*	908	908	908	908	908	908	908

Standard errors in parentheses; ***, **, or * indicates significance at 1, 5, and 10% levels, respectively.

### 5.5 Continuity tests of control variables

The test results displayed in Table 4 disclose that the wind force, the highest temperature, the lowest temperature, and the holidays were essentially stable in the periods before and after the execution of the government environmental audit and no significant changes were noted; however, the precipitation significantly increased. Theoretically, an increase in precipitation is conducive to the reduction of air pollution, primarily because rain can wash and remove air pollutants (24). The average annual rainfall in Lanzhou is less than 300 mm but the average annual evaporation is as high as 1,800–2,200 mm (25). Precipitation significantly increased but the surge was trivial compared to the evaporation. Therefore, the rise in precipitation did not alter the overall arid environment of Lanzhou, and its effect on reducing air pollution was fairly limited. Finally, the precipitation significantly increased by 0.241% after the audit pilot from a statistical perspective. Nevertheless, this relatively small shift obviously could not have greatly ameliorated Lanzhou’s air quality. In general, none of the control variables evinced a substantive jump, and therefore, none could affect the results of the model. It was thus further confirmed that the air quality enhancement was indeed evoked by the government environmental audit.

**Table 4:** Regression results of the control variable continuity tests.

Controlling variables	(1)	(2)	(3)	(4)	(5)
	h_temp	l_temp	Wind	Holiday	Rain
Mean value	−3.050	2.314	−0.286	0.246	0.241***
Standard deviation	(1.9049)	(1.4798)	(0.2608)	(0.3266)	(0.0863)
*N*	908	908	908	908	908

The polynomial of all the controlling variables selects the 7th-degree term.

Standard errors in parentheses; ***, **, or * indicates significance at 1, 5, and 10% levels, respectively.

### 5.6 Regression without control variables

As a local randomized experiment, no significant difference was noted in the control variables of regression discontinuity, indicating that the control variables on both sides of the cutoff point evinced the characteristics of randomness. Therefore, the consistency of the regression discontinuity estimators remained unaffected by the addition of control variables. The previously displayed regression discontinuity results were obtained after the addition of control variables. Therefore, the regression discontinuity results were subsequently scrutinized without adding control variables. Table 5 presents the results of RDD with and without control variables, respectively. The significance and coefficient of the regression discontinuity results without the control variables changed compared to the regression discontinuity results after the control variables were added but the variation was minor. In fact, the regression discontinuity results of the explained variable AQI decreased after removing the control variables, further exhibiting that the execution of the government environmental audit did, indeed, decrease air pollution. Hence, the control variables exerted a modest influence on the regression results of each cutoff point, and the results of each regression discontinuity also satisfied expectations.

**Table 5:** Regression results without and with control variables.

Variable	(1)	(2)	(3)	(4)	(5)	(6)
AQI	PM2.5	PM10	SO2	NO2	CO	O3
Including control variables	−59.64**	−37.25**	−90.73**	−18.60**	−27.31**	−0.730**
(29.0452)	(13.2026)	(43.2939)	(6.4465)	(6.8763)	(0.2912)	(4.0542)
Excluding control variables	−64.39*	−36.89**	−87.06**	−26.95***	−28.24***	−1.079**
(32.8685)	(12.8019)	(43.1807)	(9.5106)	(8.6893)	(0.3698)	(4.8588)
*N*	908	908	908	908	908	908

Standard errors in parentheses; ***, **, or * indicates significance at 1, 5, and 10% levels, respectively.

The results in Table 5 indicate that the coefficients and significance levels did indeed change after adding and excluding control variables. The addition of control variables may reduce residual variability in the regression model, thereby improving estimation accuracy. This improvement in accuracy may manifest as changes in coefficient estimation values and an increase or decrease in significance levels. If variables related to both explanatory variables and treatment effects are omitted from the model, these omitted variables may lead to estimation bias. Adding these omitted variables as control variables may reduce this bias, thereby altering the estimated coefficient values and significance levels.

This study utilizes daily registered pollutant data from Lanzhou between January 2014 and June 2016 to establish a regression discontinuity model aimed at evaluating the administrative effects of the government environmental audit pilot on air pollution. The findings indicate that the implementation of government environmental audits significantly improves air quality. Specifically, the audit pilot led to a notable enhancement in the overall air quality index (AQI) and resulted in varying degrees of reduction for pollutants such as PM_2.5_, PM_10_, SO_2_, NO_2_, and CO. Among these, PM_10_ exhibited the most substantial decrease, followed by PM_2.5_, SO_2_, and NO_2_, while CO showed only a minor reduction.

The differing treatment effects of various pollutants can be attributed to their distinct sources and characteristics. PM_2.5_ and PM_10_ are both types of inhalable particulate matter with similar origins, primarily arising from the combustion of fossil fuels, biomass, and dust from roads and construction activities. However, PM_10_ primarily results from the direct emission of pollutants, whereas PM_2.5_ can also be formed from the secondary transformation of gasses like nitrogen oxides and sulfur dioxide. Consequently, controlling pollution sources effectively reduces PM_10_ levels more significantly than PM_2.5_.

The reduction of PM_2.5_ levels is influenced by the emission intensities of SO_2_ and NO_2_, suggesting a coupling effect between these pollutants. This dynamic results in a more pronounced decrease in PM_10_ compared to the lesser decreases observed for PM_2.5_, SO_2_, and NO_2_. CO, primarily emitted from fuel combustion and vehicle exhaust, remained a significant air pollutant, exhibiting only a slight decline. This indicates a limited impact of government audits on controlling emissions from fuel combustion and vehicular discharge. Interestingly, the implementation of the audit pilot did not lead to a reduction in ozone concentrations. Instead, O_3_ levels increased significantly following the audit pilot. Robustness tests confirmed these findings, highlighting the challenges associated with managing O_3_ emissions. As such, it is crucial for relevant authorities to intensify their focus on O_3_ management and implement effective strategies to mitigate its impact.

The unique topography of Lanzhou, characterized by a relatively enclosed basin, limits air circulation and contributes to stable air quality. This environmental context supports the reliability of the study’s results, as Lanzhou’s atmospheric conditions are less influenced by external air. Consequently, Lanzhou should prioritize managing air pollution sources such as industrial emissions, coal burning, dust, and vehicle exhaust. A coordinated approach to addressing multiple pollutants and their diverse sources is essential, alongside ongoing efforts to the emission of CO and accelerate the regulation of O_3_.

## 6 Conclusion

This study evaluates the effectiveness of the Lanzhou Municipal Government Environmental Audit Pilot Project in controlling air pollution through empirical analysis. The study also found that there are differences in the control effects of different pollutants. PM_10_ and PM_2.5_, as inhalable particulate matter, have significantly reduced concentrations, but the decrease in PM_2.5_ is lower than that of PM_10_, which may be related to their formation mechanisms and sources. The small reduction in CO indicates that the audit has limited impact on fuel combustion and vehicle exhaust emissions. It is worth noting that government environmental audits did not reduce O_3_ concentration, but instead showed a certain degree of increase. This highlights the complexity and challenges of O_3_ governance, which require special attention and strengthened management measures. The implementation of government environmental audits has significantly reduced the air quality index (AQI) and other major pollutants (PM_2.5_, PM_10_, SO_2_, NO_2_, CO) concentrations in Lanzhou City. Specifically, AQI has decreased by 59.64 units (significant at the 1% level), and the concentrations of PM_2.5_, PM_10_, SO_2_, NO_2_, and CO have also significantly decreased. However, for O_3_ concentration, there was a significant increase of 25.54 units after the audit implementation, which may be related to the complex generation mechanism of O_3_ and meteorological conditions. Overall, government environmental audits have played a positive role in improving air quality in Lanzhou city. Before and after the government environmental audit, control variables such as wind speed, maximum temperature, minimum temperature, and holidays remained stable, while precipitation significantly increased (0.241%, with a significance level of 1%). Regardless of whether control variables are included, the negative impact of government environmental audits on air quality and its major pollutants is significant, indicating that audits effectively reduce air pollution. Specifically, when the control variable is included, AQI decreases by 59.64 units, while when the control variable is not included, it decreases by 64.39 units, and the difference between the two is not significant.

## Data Availability

The original contributions presented in the study are included in the article/supplementary material, further inquiries can be directed to the corresponding author.

## Footnotes

^1^ http://www.tianqihoubao.com
