# Corrigendum: Do government environmental audits reduce air pollution? Evidence obtained from Lanzhou

**DOI:** 10.3389/fpubh.2025.1614279

**Published:** 2025-05-16

**Authors:** Tianyou Chen, Ji Li

**Affiliations:** ^1^China Center for Special Economic Zone Research, Shenzhen University, Shenzhen, China; ^2^Research and Development Office, Shenzhen Polytechnic University, Shenzhen, China

**Keywords:** government environmental audit, air quality, regression discontinuity design, environmental protection policies, air pollution

In the published article, there was an error in the **author list** and author “Tianyou Chen” was erroneously listed as the corresponding author, whereas Ji Li is the actual corresponding author (Email: liji@szpu.edu.cn). The corrected author list appears below.

“Tianyou Chen^1^ and Ji Li^2*^

^1^China Center for Special Economic Zone Research, Shenzhen University, Shenzhen, China

^2^Research and Development Office, Shenzhen Polytechnic University, Shenzhen, China”

^*^ **Correspondence:**

Ji Li

liji@szpu.edu.cn

The authors apologize for this error and state that this does not change the scientific conclusions of the article in any way. The original article has been updated.

